# Determination of the Shear Modulus of Pine Wood with the Arcan Test and Digital Image Correlation

**DOI:** 10.3390/ma14020468

**Published:** 2021-01-19

**Authors:** Piotr Bilko, Aneta Skoratko, Andrzej Rutkiewicz, Leszek Małyszko

**Affiliations:** Department of Mechanics and Building Structures, Institute of Geodesy and Civil Engineering, Faculty of Geoengineering, University of Warmia and Mazury in Olsztyn, 10-719 Olsztyn, Poland; aneta.skoratko@uwm.edu.pl (A.S.); andrzej.rutkiewicz@uwm.edu.pl (A.R.); leszek.malyszko@uwm.edu.pl (L.M.)

**Keywords:** digital image correlation, Arcan shear test, wood, orthotropic shear modulus, elastic-plastic material, finite element method

## Abstract

Arcan shear tests with digital image correlation were used to evaluate the shear modulus and shear stress–strain diagrams in the plane defined by two principal axes of the material orthotropy. Two different orientation of the grain direction as compared to the direction of the shear force in specimens were considered: perpendicular and parallel shear. Two different ways were used to obtain the elastic properties based on the digital image correlation (DIC) results from the full-field measurement and from the virtual strain gauges with the linear strains: perpendicular to each other and directed at the angle of π/4 to the shearing load. In addition, the own continuum structural model for the failure analysis in the experimental tests was used. Constitutive relationships of the model were established in the framework of the mathematical multi-surface elastoplasticity for the plane stress state. The numerical simulations done by the finite element program after implementation of the model demonstrated the failure mechanisms from the experimental tests.

## 1. Introduction

**Figure 1:**
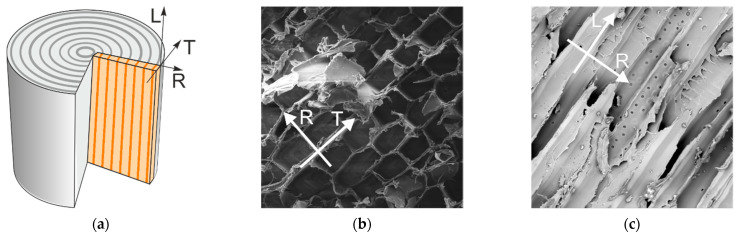
The wood material axes on a log view (**a**); scanning electron microscope photograph of a RT plane at 746 times magnitude (**b**); and scanning electron microscope photograph of a LR plane at 533 times magnitude (**c**).

Wood is an organic, naturally grown material and is commonly used for creating all kinds of goods and structures in many branches of industry. Softwood, which is mainly used for structural and load-bearing purposes in civil engineering, at a micro-scale level, is built from axial tracheids connected between themselves by a lignin matrix. The tracheids (see Figure 1b, which shows a tracheid in a perpendicular cut) are long, thin cells organized in a way that their length is parallel to the length of the log and are the main source of the wood strength. They are “glued” by lignin at the edges of its cell walls and create the annual rings. The micro-scale built is a basis for understanding the macro behavior and strength of clear wood (i.e., a material considered as without flaws, e.g., resin pores or knots), which is generally high in the longitudinal direction (denoted as L), where the tracheid’s generate strength, and low in the two other directions, i.e., radial and tangential (denoted by R and T, respectively), where the lignin matrix has lower mechanical properties (see Figure 1b,c for direction denoting). Therefore, the weakest mechanical properties of wood are those at the direction normal to the fibers during tension (i.e., R and T) or shear along the longitudinal direction (i.e., L), causing a rupture between annual rings. Exposure to these types of stresses easily leads to cracking, which usually forms along the grain direction, choosing the path of least resistance.

The heterogeneity, orthotropy and high variability of naturally grown wooden materials makes both modeling and experimental investigations challenging. The natural origin of wood, being its major advantage, is also a major obstacle in the advancement of wood research. The lack of manufacturing control of the material properties, as is possible in artificial materials, as well as the complicated internal structure of wood from micro- to macro-scale, gives a substantial level of uncertainty in the interpretations of test results and model approaches [1,2]. The clear wood is often treated mechanically as an orthotropic material with three specified material axes (Figure 1a), i.e., the longitudinal (L), radial (R) and tangential (T) ones. Its macroscopic behavior originates from its microscopic structure—fibers—and their directional arrangement (Figure 1b,c). Moreover, in one annual ring, the mechanical characteristics are different due to early (spring) and late (autumn) growth characteristics of this ring. The earlywood grows more quickly and is weaker, which is in opposition to the latewood. In general, it rises the additional issue of material inhomogeneity, which is typical for materials of natural origin (e.g., wood and soil). It is also an issue where is the limit of considering wood as a homogeneous material, which is a common engineering practice. This is important especially while examining the shearing of LR plane of orthotropy with the direction of the shearing load (P) parallel with the longitudinal material axis (L). A crack may lie in the LR plane and may propagate in one of two directions, from which more practical importance has the one along the lower strength path parallel to the grain. This system of propagation, where L is the direction in which the crack propagates (the LR,L system), will predominate as a result of the low strength and stiffness of wood perpendicular to the grain. The opposite of this is the LR,R system, where the crack propagates in the direction R.

In general, the experimental determination of the wood behavior in shear has always been influenced by difficulties in obtaining a pure and uniform shear state. This issue resulted in many different experimental methods: the Arcan test, off-axis tests, Iosipescu test, four-point bending test, etc. [3]. Numerical simulations, performed for tests, usually indicate a combination of normal and shear stresses, making difficult to interpret the pure shear behavior. Among the mentioned methods, the Arcan shear test [4] is considered to create a rather uniform and pure state of shear stress among the critical cross section. The main problem arises with boundary conditions, which are strongly dependent on the type of specimen fixture and the distance to the critical cross section, and can influence the behavior of the specimen. The Arcan test on wood has been studied (see, e.g., [5,6,7,8,9]), where strains are measured using strain gauges [6,9], with video extensometers [5] or not measured at all [7]. Tests with the digital image correlation were used by the authors of [8,10]. The work in [6] is of significant importance because it gives the shear constants in all three material planes at two load-to-material axes directions, which are rarely obtained due to the labor-intensive nature of such tests.

**Figure 2:**
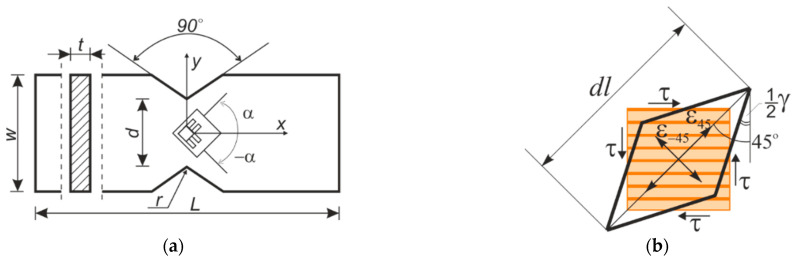
The standard shear modulus test specimen-strain gauge T-rosette and a way of measurement of the shear angle (**a**); and a state of pure shear (**b**). All dimensions are in millimeters (mm) unless otherwise noted.

Although shearing tests have been performed using many techniques, the increasement in measuring technology makes it possible today to gather more information and obtain new results. Technology of the digital image correlation (DIC) enables recording and analyzing the whole surface of the specimen, on both sides [11]. The possibilities can be shown in a simple example presented in Figure 2. Measurements using strain gauge T-rosettes enable measuring two values at one “point” (this point is distributed along the strain gauge grid length) (Figure 2a) [7]. The DIC enables measuring the displacements (and further calculate strains) of approximately 2400 points in the observed area. However, the accuracy of the system is still studied.

In this paper, apart from the experimental studies described in Section 2, the results of numerical simulations of the performed tests are also presented in Section 3. The simulations were carried out using the own orthotropic material model of clear wood, as discussed in [12]. Three basic failure mechanisms in plane stress are distinguished in the model: failure due to tensile, compressive and shear stresses. The composite failure criterion consists of three analytical expressions, each of them being a limit equilibrium condition of the material in a complex stress state.

## 2. Experimental Studies

### 2.1. Background Theory

The 2D strains components, the normal strains $\varepsilon_{x},\varepsilon_{y}$ and the shear strain $\varepsilon_{xy}$, are directly calculated in the software of the digital image correlation system [13] from the symmetrical material stretch tensor U:

(1) $$U=\sqrt{F^{T}F}=\left(\begin{array}{cc} 1+\varepsilon_{x} & \varepsilon_{xy}\\ \varepsilon_{xy} & 1+\varepsilon_{y} \end{array}\right),$$

where F is the deformation gradient. The shear angle $\gamma_{xy}$ without the rigid rotation is calculated as:

(2) $$\gamma_{xy}=\gamma_{x}+\gamma_{y}=\arctan\left(\frac{\varepsilon_{xy}}{1+\varepsilon_{x}}\right)+\arctan\left(\frac{\varepsilon_{yx}}{1+\varepsilon_{y}}\right),$$

where $\gamma_{x}$ and $\gamma_{y}$ are the corresponding shear angles of the two sides of the deformed elemental square (see also [7]). Note that the software gives the strains and angles at the certain points referring to an arbitrary coordinate system (x,y).

The constitutive law of linear elasticity for the orthotropic material is determined by nine independent material parameters because of the strain and stress tensors symmetries and the existence of the elasticity energy function. As is the case for the orthotropic material, the formulas of Hooke’s law depend on the orientation of the coordinate system in a reference to the principal axes of material symmetry, i.e., the axes of orthotropy. The shear moduli in the frame of reference aligned with the orthotropic axes, so-called the technical moduli, can be calculated independently of other material constants as:

(3) $$G_{LR}=\frac{\tau_{LR}}{\gamma_{LR}},\;G_{LT}=\frac{\tau_{LT}}{\gamma_{LT}},\;G_{RT}=\frac{\tau_{RT}}{\gamma_{RT}},$$

where $\tau_{LR},\;\tau_{LT},\;\tau_{RT}$ are the shear components of the stress tensor in the planes LR, LT, RT, respectively, and $\gamma_{LR},\;\gamma_{LT},\;\gamma_{RT}$ are the corresponding shear angles.

To determine the shear angle γ in the chosen plane, the following engineering geometrical considerations is used additionally. Let us consider an infinitely small element in the plane LR, which is in a state of pure shear (Figure 2b). When shearing, the right angles change by the value $\gamma =\gamma_{LR}$. The one diagonal is then lengthened with the strain $\varepsilon_{45}$ and the second diagonal shortens with the strain $\varepsilon_{-45}$ and:

(4) $$\tan\left(\frac{\pi }{4}-\frac{\gamma }{2}\right)=\frac{dl(1+\varepsilon_{-45})}{dl(1+\varepsilon_{45})}\approx \frac{1-\gamma /2}{1+\gamma /2},$$

where dl is the diagonal length of the element. From (4), we get:

(5) $$\gamma =\frac{2(\varepsilon_{45}-\varepsilon_{-45})}{2+\varepsilon_{45}+\varepsilon_{-45}}.$$

To derive the engineering shear angle γ in the DIC system, the construction of the virtual strain gauges is required in the same way as for two-element strain gauge rosettes, e.g., for a 10 × 10 mm^2^ square, where $\varepsilon_{45},\varepsilon_{-45}$ are the linear strains, perpendicular to each other and directed at 45° angle to the shearing load.

### 2.2. Specimens, Equipment and Methods

**Figure 3:**
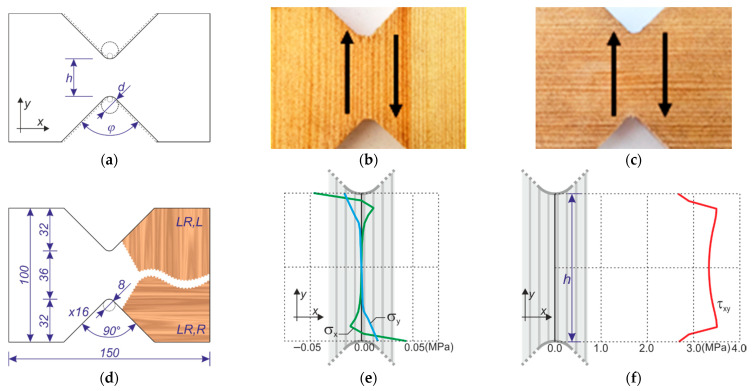
The specimens: (**a**) dimensions subjected to variation; (**b**) LR,L orientation; (**c**) LR,R orientation; (**d**) geometry of specimen used for shear tests; and (**e**,**f**) stress distributions in the middle section for LR,L specimens. All dimensions are in millimeters (mm) unless otherwise noted.

The dimensions of the specimen were preliminarily defined by the basic numerical tests on several different configurations of the critical cross-section height and curvature. Nine different shapes were modeled checking stress distribution by means of the finite element method. The dimensions modified were: the shear area height h→{36,40} mm, the initial diameter d→{4, 8, 12} mm and the inclination angle φ→{45°, 60°, 75°, 90°} of the cutting lines (see Figure 3a). The aim was to achieve a pure shear state in the middle section of the specimen; hence, it was sufficient to adopt an isotropic material and perform the simplified analysis only in the elastic range. The most satisfactory results were obtained for the following dimensions: h=36 mm, d=8 mm and φ=90° (all dimensions are shown in Figure 3d). The obtained tangential stress distribution was characterized by low variability with practically zero values of the associated normal stresses. The stress distributions for the middle cross-section are shown in Figure 3e,f.

**Figure 4:**
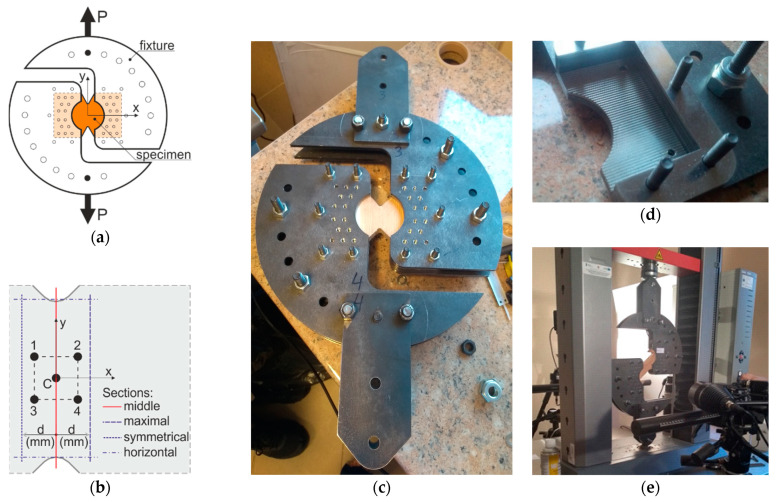
The Arcan fixture: (**a**) scheme; (**b**) the explanation of measuring points/sections; (**c**) physical fixture; (**d**) distances and tooth plates; and (**e**) experimental setup with the DIC system.

Storage and processing of wood specimens were performed in normal conditions of 65% relative humidity and temperature of 20 °C. The pre-specimens were firstly cut from 16 mm planks (planks cut from the central part of the log) of pine wood (*Pinus sylvestris* L.) with rectangular dimensions of 100 mm × 150 mm concerning two perpendicular directions of LR plane (in Figure 3b,c, the arrows show the shearing directions with respect to the material axes). Further, the notches were made using a milling-machine with a down spindle and a saw to create the required shape (Figure 3d). The tests were performed immediately after the transportation to the testing facility and prepared for the DIC measurements. Therefore, the surface of the specimens was sprayed using a black aerosol can to create a more stochastic pattern. Since wood has an inhomogeneous surface, there little paint was needed. Further, the specimens were inserted into the Arcan fixtures (Figure 4). The fixture dimensions and shape were designed by the authors and water cut from an 8mm thick stainless-steel plate. The fixture elements were connected by 8 and 12 mm steel screws with steel plates used as distances, while the specimens were tightened by steel tooth plates (Figure 4d).

The test was performed using the 10 kN nominal force universal testing machine, equipped with the Arcan fixture. A digital image correlation system [13] was used to obtain the full-field displacement distribution and visualize the shear strain uniformity. The testing machine gave information on the forces, while the DIC system on the shear angles. The preparation of the DIC system started with choosing the calibration object, here the manufacturer’s CP90/20 was used, which allowed measuring an area between 78 × 65 mm^2^ and 130 × 105 mm^2^ (final area was approximately 85 × 70 mm^2^). Afterwards, the typical system calibration was performed. The software options were chosen as: facet size 19 × 19 pixels and faced step (distances between facets) 15 × 15 pixels, as proposed by the manufacturer. The DIC used in this experiment was composed of two 5Mpix cameras (resolution 2448 × 2050). The starting points for calculations were chosen, as recommended, at areas where the displacements were minimal (typically, the lower left part of the specimen). The experimental setup of the DIC system is shown in Figure 4e.

Twelve specimens were tested. Six of them were oriented so that the shear direction was parallel to the L axis (LR,L-specimens) and the other six with the shear direction parallel to the R axis (LR,R-specimens). Both tests were tracked automatically by displacement with a constant value of 0.35 mm/min and an initial force of 130 and 70 N for the LR,L and LR,R specimens, respectively. The ultimate forces were taken from the testing machine at the moment of failure (LR,L specimens) and at the moment of first horizontal crack (LR,R specimens). The values of ultimate shear angle and shear modulus were calculated for central point of the cross-section (indexed “c”) (Point C in Figure 4b and Expression (2)) and for Points 1–4 presented in Figure 4b (indexed as “g”) (from the virtual gauges located on the diagonals of the central square of 10 × 10 mm^2^ and Expression (5)). The ultimate shear angle values were taken at the moment of failure.

The calculations of the LR shear moduli were performed according to Formula (3) with the shear angle obtained from (2) for the $G^{c}$ moduli and with the shear angle obtained from (5) for the apparent $G^{g}$ moduli. The shear modulus was determined as a secant modulus in the range between 25% and 50% of the maximal external force $P^{ult}$ in each specimen. Such values were chosen due to very small shear angles for the measuring system resolution (initially 10–40% of the maximal force was considered). The expression for calculating the modulus from the experimental results can be written as follows:

(6) $$G=\frac{\Delta \tau }{\Delta \gamma }=\frac{\tau^{50\%P}-\tau^{25\%P}}{\gamma^{50\%P}-\gamma^{25\%P}},$$

where the shear angles are taken as from Equation (2) or (5) for $G=G^{c}$ and $G=G^{g}$, respectively. The nominal shear stresses τ were computed as a ratio between the force P and nominal cross-section $A_{nom}$, i.e., $\tau =P/A_{nom}$.

The shear angle maps were generated by the software for stages just before the failure i.e., rupture for the LR,L specimens and first horizontal crack for the LR,R specimens. Three different vertical sections were prepared to provide complete information on the distribution of strains along the cross-section of the LR,L specimens, as shown in Figure 4b: the “middle cross-section” (red continuous line), the “maximal cross section” (blue dashed line) and the “symmetrical cross section” (blue dashed line). For the Specimens LR,R, two sections of different lengths were used: the “middle cross-section” and the horizontal blue dash-dot one.

In addition, for the obtained τ−γ relationship, a linear approximation of results was done by the use of the least square method. The method finds the best fit, in this case the shear moduli itself, which is a tangent of the angle between the linear fit and the horizontal axes. The shear angles were taken from Expression (2) and the fitting range was 25–100% of the maximal external force $P^{ult}$ (rupture in LR,L direction and first crack in the LR,R direction).

### 2.3. Results

**Figure 5:**
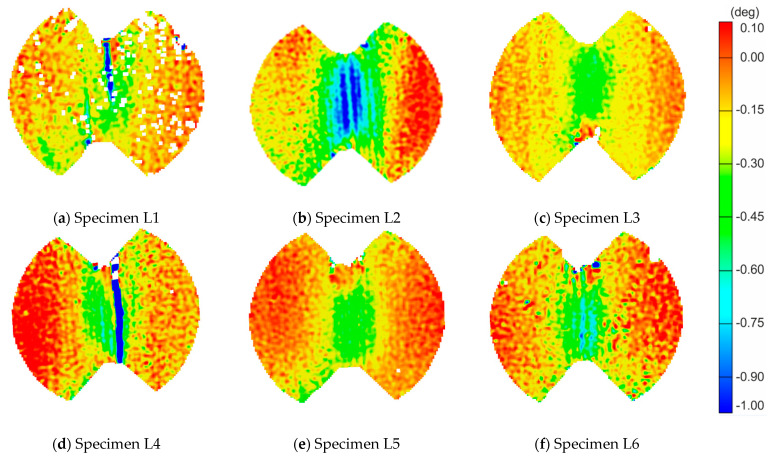
The shear angle maps just before the failure for each LR,L-type specimen: (**a**–**f**) for each of the samples from L1 to L6 respectively.

**Figure 6:**
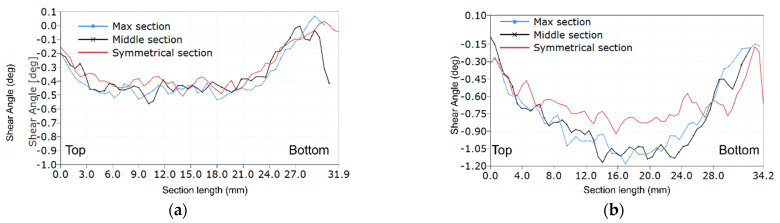
The shear angle plots along critical sections for LR,L-type specimens: (**a**) Specimen L3; and (**b**) Specimen L2.

The post-processing of strain values was performed in the DIC software. Maps of the shear angle and charts at the moment just before failure for all six LR,L specimens are shown in Figure 5 and Figure 6, respectively.

The LR,L results in the maps of shear angle show different failure sections. The maps for Specimens L1 (Figure 5a) and L4 (Figure 5d) clearly show that the failure arises not in the central cross-section as expected, but next to it. This is generated due to the material inhomogeneity, where most likely a wider strip of earlywood was defining the path of failure. The strains in Specimens L2–L6 are concentrated around the central part of the specimen. In addition, Specimens L3, L5 and L6 present more uniform strain distribution among the others. Figure 6 shows the distribution of the shear angle along vertical sections. In general, the distribution of deformations in the central part is close to parabolic. However, in the case of Specimen L3, there is a more even distribution across the width as compared to sections from the symmetrical to max section (Figure 6a). In Specimen L2 (Figure 6b), the greater values of the angle, and hence the greater stress intensity, are on the right side of the central axis.

**Figure 7:**
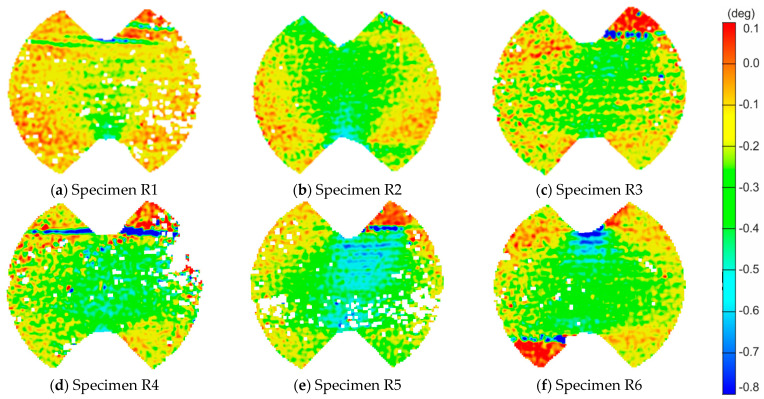
The shear angle maps just before the failure for each LR,R-type specimen: (**a**–**f**) for each of the samples from R1 to R6 respectively.

**Figure 8:**
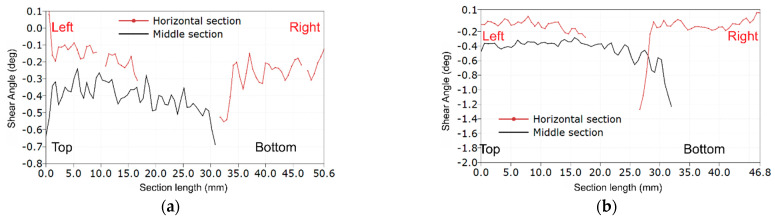
The shear angle plots along critical sections for LR,R-type specimens: (**a**) Specimen R2; and (**b**) Specimen R6.

In Figure 7 and Figure 8, we can find similar information for LR,R specimens. Figure 7 shows the deformation maps. The failure crack occurs horizontally along a line taken from the edge of the weakened central section (see Specimens R1, R3, R4 and R6 in Figure 7). In Figure 8, an increase in the value of shear strains can be noticed in the vicinity of the initial and end coordinates corresponding to the edges of the specimen (vertical middle section, black solid line). The red line of the results for the horizontal section also shows the highest values in the middle, i.e., near the critical edge.

The obtained results on shear moduli, ultimate strength and strain are shown in Table 1 for the LR,L specimens and in Table 2 for the LR,R specimens. The apparent shear modulus $G^{g}$ and apparent ultimate shear angle $\gamma^{g,ult}$ from Expression (5) the coefficient of variation (COV) did not exceed 24%, which are quite big but at an acceptable level in wood. The same values calculated by (2) show acceptable COVs for the modulus ($G^{c}$) and a moderately high value for the ultimate shear angle ($\gamma^{c,ult}$).

The values of shear strength for the LR,L specimens had a mean value of 4.4 ± 0.7 MPa, with a standard deviation of 0.85 MPa and a COV of 19.2%. The ultimate force had a mean value of 2448 ± 404 N, a standard deviation of 492 N and a COV of 20.1%. The apparent shear modulus had a value of 392 ± 55 MPa, a standard deviation of 67 MPa and a COV of 17.1%. In linear elastic orthotropic theory, the LR and RL moduli are considered as equal. The value of the modulus obtained from LR,L specimens is relatively low. The reason lies in the high variability of the measured shear angles: a COVs of 34% from the Expression (5) and 54% for the Expression (2). Hence, they were considered nonrealistic results and disregarded.

**Figure 9:**
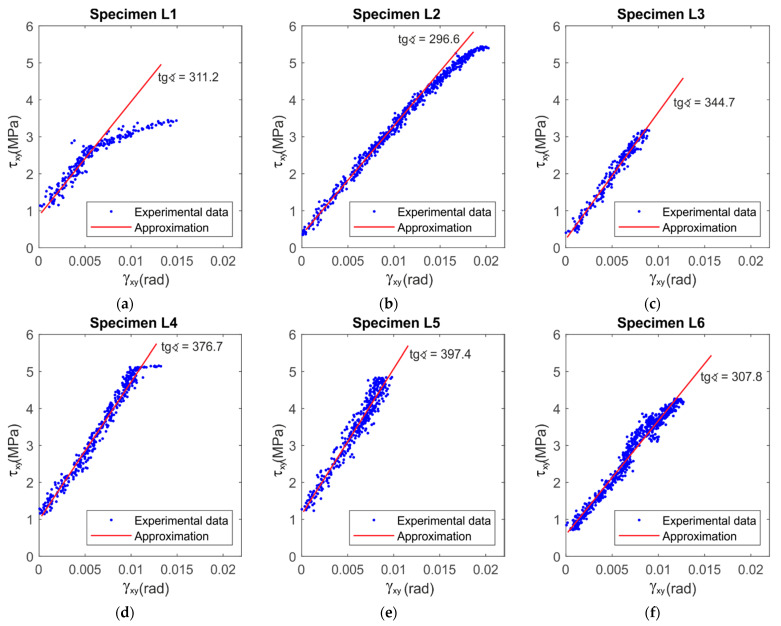
Charts of the relationship of shear stress–shear angle at the central point for LR,L specimens with their linear approximation: (**a**–**f**) for each of the samples from L1 to L6 respectively.

**Figure 10:**
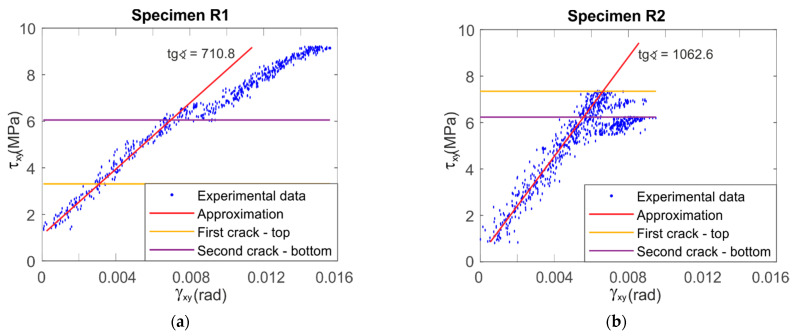

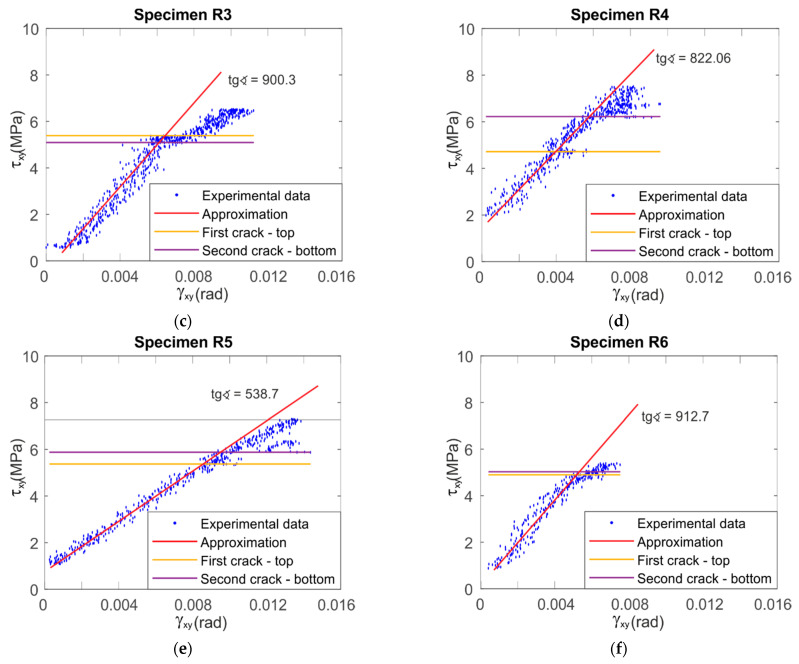
Charts of the relationship of shear stress–shear angle at the central point for LR,R specimens with their linear approximation: (**a**–**f**) for each of the samples from R1 to R6 respectively.

Figure 9 and Figure 10 show the stress–strain relationships for LR,L and LR,R specimens, respectively. The shear angles were taken from Expression (2) for the central point. The red lines show a linear fit in the range of results from 0.25 of the ultimate force up to the moment of failure. The LR,L specimens present a brittle failure (Figure 9), while LR,R present a linear behavior up to the first crack. After the crack, the specimen is changing the configuration and the specimen is slightly rotating and deforming, which is why the experimental points seem to lay on each other, due to stress loss after the crack, especially on the specimens shown in Figure 10b,e. The obtained LR,L average modulus of shear is approximately 350 MPa, while that of LR,R is approximately 840 MPa. These results are similar to those shown above in Table 1 and Table 2. This confirms the validity of the previously adopted methods.

The results of the LR,L specimens shows a very low average modulus of shear of approximately 400 MPa, while the LR,R modulus is approximately 800 MPa. It may be caused by a relatively great ratio of earlywood-to-latewood width in the used wooden specimens. This ratio depends only on the growth conditions of the tree. The experimental scheme causes that, in the LR,L direction, the earlywood becomes dominant in material behavior as the more susceptible material part, whereas, in the LR,R direction, both material parts, early- and latewood, are working simultaneously.

Another issue lays in system accuracy. Consider the noise of the system; analyzing the chart of the L3 specimen in Figure 6a, successive points from each line can differ even by 25%. These values are calculated using Expression (2), which uses a small area to gather information called facets, i.e., a square defined in pixel size in the software (in this case 19 × 19 pixels) and corresponding true dimensions of approximately 0.5 × 0.5 mm^2^ (in this particular case). For homogeneous fields with large strain values, such as plastic flows in steel or displacements of parts, this is sufficient. However, it may be more complex to calculate a very inhomogeneous strain field, where early- and latewood particles are mixed, and strains are very low. Therefore, we can consider the maps as a qualitative source of information. However, this does not forbid the quantitative analysis—the noise of the results is relatively high but can be reduced to some level by averaging results, as well as considering using a greater calculating area—of the aforementioned facets, where it is possible to increase such fields to dimensions of even 50 × 50 or 100 × 100 pixels to reduce the noise at a cost of calculation time, which was already proved by some experimental works. Moreover, the displacement measuring accuracy can be easily increased by using a greater length of the measuring base. That is why the apparent values of the moduli and ultimate shear angles are reliable—the values are calculated using a 10 × 10 mm^2^ square and the displacement of these points is seen by the system well.

## 3. Numerical Simulations of Failure Mechanisms in the Tests

The numerical simulations of timber shearing in the Arcan test (Figure 4a [8]) with the failure modes and mechanisms are the purpose of this section after the implementation of the own continuum structural models from [12,14] into the commercial finite element code [15]. The Arcan test is considered to create a rather uniform and pure state of shear stress among the critical cross section. However, an appropriate constitutive model and the analysis by means of the finite element method allow more detailed insight into the sequence in which crack zones develop. The constitutive relationships of the model have been established in the framework of the mathematical elastic–plastic theory of small displacements. The model is based on the three orthotropic failure criteria that were earlier proposed by Geniev and next incorporated into the plasticity condition as the composite yield surface [16,17]. This orthotropic failure criteria can be regard as generalization of the well-known an isotropic maximum principal stress criterion of Rankine extended to the tension and compression anisotropic regimes and the Mohr–Coulomb strength criterion for the shear regime. They have the following forms in the plane state of stresses and in the frame of reference coincided with the axes of the principal stresses:

(7) $$\left(\frac{\cos^{2}\varphi }{Y_{t1}}+\frac{\sin^{2}\varphi }{Y_{t2}}\right)\sigma_{1}+\frac{\sigma_{1}\sigma_{2}}{Y_{t1}Y_{t2}}+\left(\frac{\sin^{2}\varphi }{Y_{t1}}+\frac{\cos^{2}\varphi }{Y_{t2}}\right)\sigma_{2}-1=0\;,$$

(8) $$\left(\frac{\cos^{2}\varphi }{Y_{c1}}+\frac{\sin^{2}\varphi }{Y_{c2}}\right)\sigma_{1}+\frac{\sigma_{1}\sigma_{2}}{Y_{c1}Y_{c2}}+\left(\frac{\sin^{2}\varphi }{Y_{c1}}+\frac{\cos^{2}\varphi }{Y_{c2}}\right)\sigma_{2}+1=0\;,$$

(9) $$\begin{array}{l} \sigma_{1}^{2}-2(1+2\mu^{2})\;\sigma_{1}\sigma_{2}+\sigma_{2}^{2}+2\mu \;(C_{11}+C_{22})(\sigma_{1}+\sigma_{2})+\\ +2(C_{11}-C_{22})\;(\left|\sin2\varphi \right|-\mu \cos2\varphi )(\sigma_{1}-\sigma_{2})-4C_{11}C_{22}=0\;, \end{array}$$

where φ denotes an angle between the axis of the first principal stress and the first axis of orthotropy.

The four uniaxial strength parameters $Y_{\Delta i},\;i=1,2$ appear in the Rankine-type criteria described by Formulas (7) and (8), which are obtained from the two tensile tests (Δ=t) and two compressive tests (Δ=c) in the directions of the first and second axes of orthotropy, respectively. In Formula (9), we can find the parameter of internal friction μ and the shear strength parameters $C_{11}$ and $C_{22}$ obtained from the tests with the predetermined shear failure planes which are coincided with the orthotropy axes. Three different characteristic values of the shear stress can be calculated from Criterion (9) for the stress state $\sigma_{1}=-\sigma_{2}$ and the angle $\varphi =0^{0},\;45^{0},\;90^{0}.$ The value of the shear stress for the angle $\varphi =\;45^{0}$ is of the particular interest, because the direction of the shearing then coincides with the orthotropic axes. This shear stress can be helpful in setting the strength parameters in the numerical simulation of the experimental tests, and it is obtained from the following relationship:

(10) $$\tau_{\max}=\frac{\sqrt{(C_{11}-C_{22})+4(1+\mu^{2})C_{11}C_{22}}-(C_{11}-C_{22})}{2(1+\mu^{2})}\;.$$

**Figure 11:**
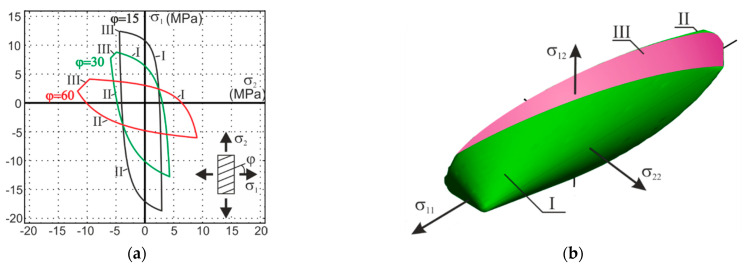
Contours of orthotropic strength criteria: (**a**) in the principal stress space; and (**b**) in the axes of orthotropy (I) and (II) are Rankine-type criterion for tension and compression regime and (III) is the Mohr–Coulomb shear failure criterion.

Contours of the failure criteria in the principal stress state are presented in Figure 11 for different values of the angle φ and in the axes of orthotropy.

### 3.1. Implementation of the Model

The more detailed discussion of the constitutive equations of the similar models and their numerical implementation into the commercial FEM system has been recently presented [18,19]. The plastic part of the strain tensor is defined by a flow rule associated with the yield function given by the plasticity (failure) criterion written in the following form:

(11) $$f_{\Delta }\left(\sigma ,\alpha_{\Delta ,in}\right)=\frac{1}{2}\sigma \cdot P_{\Delta }\cdot \sigma +p_{\Delta }\cdot \sigma -(1+K_{\Delta }\alpha_{\Delta ,in})=0$$

where $K_{\Delta }$ is a given constant plastic parameter and $\alpha_{in}$ is an internal hardening variable, hence the fourth- and second-order symmetric tensor functions $P_{\Delta }$ and $p_{\Delta }$ are dependent on the strength parameters of Criteria (7–9). The double contraction of the tensors is denoted by one dot. The plastic parameter $K_{\Delta }=0$ for the perfect plasticity, $K_{\Delta }>0$ for the hardening and $K_{\Delta }<0$ for the softening behavior [12]. Since the model consists of three yield surfaces (11), we identify the material parameters by adding the subscript index Δ, where Δ=t is assigned to the tension Condition (7), Δ=c to the compression Condition (8) and Δ=s to the shear Condition (9).

The model was implemented as the user-supplied subroutine into the FE system DIANA [15], in which the nonlinear material behavior is updating over the equilibrium step within a framework of an incremental-iterative algorithm of the finite element method with a return-mapping algorithm and a consistent tangent stiffness operator for the plane stress state. The implementation was a very demanding programming task of the subroutine USRMAT in the FORTRAN language, which is described in detail in [18,19]. The formulation of the model during the implementation was presented based on the assumption that the principal axes of orthotropy coincided with the Cartesian frame of reference for stresses and strains in finite element computations. The tensor functions $P_{\Delta }$ and $p_{\Delta }$ have then the following matrix representations for tension and compression regimes:

(12) $$p_{t}\Rightarrow \left[\begin{array}{c} \frac{1}{Y_{tL}}\\ \frac{1}{Y_{tR}}\\ 0 \end{array}\right],\;p_{c}\Rightarrow \left[\begin{array}{c} \frac{1}{Y_{cL}}\\ \frac{1}{Y_{cR}}\\ 0 \end{array}\right],\;P_{\Delta }\Rightarrow \left[\begin{array}{ccc} 0 & \frac{-1}{Y_{\Delta L}Y_{\Delta R}} & 0\\ \frac{-1}{Y_{\Delta L}Y_{\Delta R}} & 0 & 0\\ 0 & 0 & \frac{2}{Y_{\Delta L}Y_{\Delta R}} \end{array}\right]$$

and

(13) $$p_{s}\Rightarrow \left[\begin{array}{c} \frac{\mu }{C_{LL}}\\ \frac{\mu }{C_{RR}}\\ \frac{C_{LL}-C_{RR}}{C_{LL}C_{RR}}sign(\tau_{LR}) \end{array}\right],\;P_{s}\Rightarrow \left[\begin{array}{ccc} \frac{1}{2C_{LL}C_{RR}} & \frac{-(1+2\mu^{2})}{2C_{LL}C_{RR}} & 0\\ \frac{-(1+2\mu^{2})}{2C_{LL}C_{RR}} & \frac{1}{2C_{LL}C_{RR}} & 0\\ 0 & 0 & \frac{2(1+\mu^{2})}{C_{LL}C_{RR}} \end{array}\right]$$

for the shear regime. In Formulas (12) and (13), the frame of reference is denoted as $\left(x_{L},x_{R},x_{T}\right)$ and the shear strength parameter, e.g., $C_{LL}$, is obtained from the direct shear test in which the normal to the shear plane is predetermined in direction of the first axis of orthotropy.

Several tests confirmed the correctness of the proposed numerical algorithm for the anisotropic continuum. The multi-surface model enables the identification of the relevant macroscopic failure modes. The separated description of the three regimes also allows the modeling of their respective post-failure behavior with modern hardening/softening evolution laws, although an intersection of different yield surfaces defines corners that require special attention in the numerical algorithm.

### 3.2. FEM Modeling and Results

**Figure 12:**
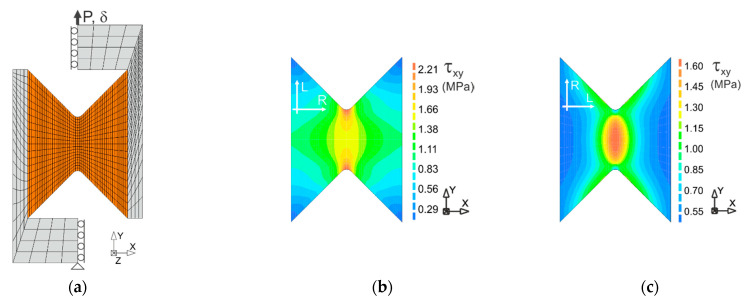
Distributions of the shear stress for different configurations of the specimens: (**a**) the finite element mesh; (**b**) Specimen LR,L; and (**c**) Specimen LR,R.

The finite element mesh was created out of 1321 nodes and 1232 elements. The geometry of FE mesh with boundary conditions is presented in Figure 12a. The type of used elements was the Q8MEM (isoparametric, cuboid eight node elements). The force was inducted by displacement of the upper arm of the fixture, so the analyses were carried out with indirect displacement control. The following material parameters were adopted based on the tests [10]: the Young’s moduli $E_{LL}=13.7$ MPa, $E_{RR}=1.1$ MPa, the shear modulus $G_{LR}=820$ MPa and the Poisson’s ratio $\nu_{LR}=0.45$. Other material parameters are presented in Table 3. When assuming the shear strength, the results from the experiments discussed above were considered. However, due to their large spread, it was decided to round the values. It should be noted that in Table 3 the yield strengths are assumed in the numerical computations, which can be different from the experimental strengths.

**Figure 13:**
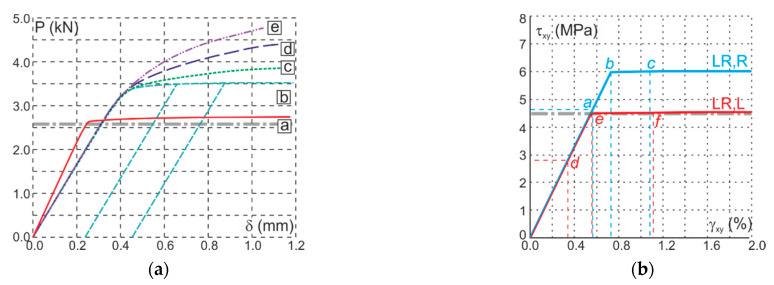
Graphs of the responses obtained from the numerical tests-description in the text: (**a**) load vs. displacement graphs; and (**b**) stress–strain curves for the middle point of the specimen.

**Figure 14:**
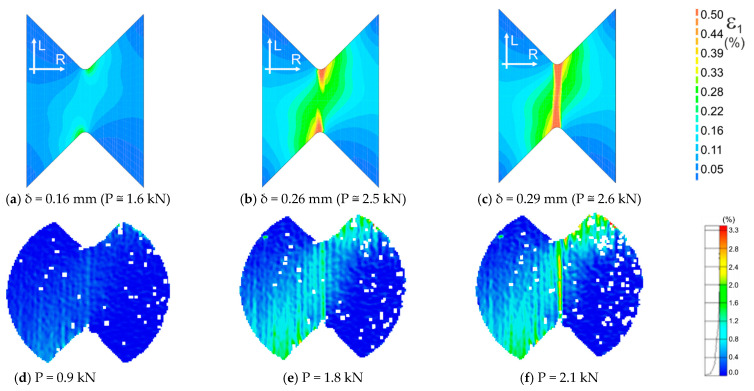
Comparison of the maps of the maximum principal strains $\varepsilon_{1}$ obtained numerically (**a**–**c**) and calculated from displacements measured by the DIC method (**d**–**f**) for LR,L orientation.

Figure 12, Figure 13 and Figure 14 present the results of the numerical simulations of the test for the specimens with different orientations—LR,L and LR,R. Figure 12 presents shear stress distributions in the elastic state for the displacement level of δ=0.1 mm. This level corresponds to values of the external force P = 1.5 kN for Specimen LR,L (Figure 13a, Line a) and P = 0.75 kN for Specimen LR,R (Figure 13a, Line b). It is seen in Figure 12 that the uniform state of shear stress occurs only in the middle of the specimens and its distributions are different depending on the orientation of the material axes.

Figure 13a shows the relationship between reaction and displacement of the upper support (P-δ) for different values of the plastic parameter. Lines [a] and [b] are for the perfect plasticity. For the comparison, Lines [c]–[e] are also shown in Figure 13a for Specimen LR,R and the hardening plasticity with *K_s_* = 10 for Line [c], *K_s_* = 50 for Line [d] and *K_s_* = 200 for Line [e]. Good agreement was found between numerical and experimental results of the ultimate external force for the perfect plasticity and Specimen LR,L—2.73 (the numerical simulation) and 2.58 kN (the experiment)—and the worse agreement for the Specimen LR,R—3.50 and 2.90 kN, respectively.

The value 2.90 kN of the ultimate external force corresponds to the moment of the first crack appearance. The second crack appears with an average load of 3.30 kN, which is closer to the numerical result. The hardening effect similar to the experimental results is visible in Figure 10 and can be easily controlled by the appropriate selection of *K_s_* parameter. Lines [c]–[e] in Figure 13a are an example of the possibilities offered by the model. The exact fit will be the subject of further research. Figure 13b shows the relationship between shear stress and strain in the central point. Red Line [a] is for Specimen LR,L and perfect plasticity (*K_s_* = 0). The obtained maximum strength was 4.50 MPa, which corresponds to the adopted value of the shear strength ($C_{RR}$). Blue Line [b] is for Specimen LR,R and perfect plasticity (*K_s_* = 0). Again, the obtained maximum strength 6.0 MPa corresponds to the adopted value of the shear strength ($C_{LL}$). The slope in the elastic range is consistent with the adopted value of the modulus. Another path to destruction has been observed. For the test in the LR,L configuration, the first active was the shear criterion, while, in the LR,R configuration, the tensile criterion was activated first. Points marked with letters *a* and *d* shown in Figure 13b correspond to the first moments of reaching the failure criterion.

**Figure 15:**
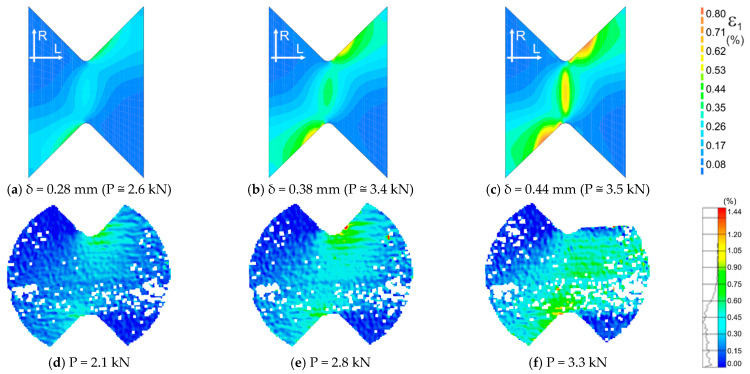
Comparison of the maps of the maximum principal strains $\varepsilon_{1}$ obtained numerically (**a**–**c**) and calculated from displacements measured by the DIC method (**d**–**f**) for LR,R orientation.

In Figure 14 and Figure 15, we can find maps of the plastic strains at different stages of the tests. The maps in Figure 14a–c correspond to the test moments marked with points *d–f* in the diagram of Figure 13b, respectively. The maps in Figure 15a–c correspond to the test moments marked with points *a–c* in the diagram of Figure 13b, respectively. The obtained mechanisms are compatible with those obtained experimentally and shown in Figure 14d–f and Figure 15d–f.

## 4. Conclusions

The paper presents experimental investigations of wood shearing in the LR plane for two different directions of loading. Twelve specimens were tested. Forces and shear angles were measured using a testing machine and digital image correlation. Some of the material constants and strengths were determined. Shear angle maps and charts for the critical cross sections are presented.

The usage of the DIC system showed that it is capable of gathering more information on the experiment than typically used measuring techniques such as strain gauges. The graphical presentation in the form of maps showed a great inhomogeneity on the specimen surface in case of the shear angles distribution.

The results on the two different directions of loading show that it may be necessary to reconsider the specimen shape and border conditions of the test to obtain the homogenized material parameters. This issue is depending to a very large ratio between earlywood and latewood among annual rings. This issue may affect the results of specimens of different species with lower early-to-latewood ratios, to a certain extent.

Our own constitutive model for the analysis of wooden structures in biaxial plane stress states, implemented into the finite element code, was used to analyze behavior of wood during shearing in the Arcan test. Experimental determination of the shear behavior has always been influenced by difficulties in obtaining a state of pure and uniform shear in test specimens. Model calibration allows adjustment to experimental results. For different specimen material axis orientations, adequate destruction mechanisms were obtained.

## Figures and Tables

**Table 1:** The results from the monotonic test of the LR,L specimens.

	$P^{ult}$	$\tau \;=\;P^{ult}/A_{nom}$	$\gamma^{g,ult}$	$G^{g}$	$\gamma^{c,ult}$	$G^{c}$
	(N)	(MPa)	(10^−3^)	(MPa)	(10^−3^)	(MPa)
Mean	2448 ± 404	4.4 ± 0.7	11.5 ± 3.6	392 ± 55	8.73 ± 3.9	470 ± 133
SD	492	0.85	4.37	67	4.75	161
COV	34.3	20.1	38	17.1	54.5	34.3

${}^{ult}$ corresponds to the maximum external force; ${}^{g}$ the values from the virtual gauges; ${}^{c}$ the values for central point of the cross-section.

**Table 2:** The results from the monotonic test of the LR,R specimens.

	$P^{ult}$	$\tau \;=\;P^{ult}/A_{nom}$	$\gamma^{g,ult}$	$G^{g}$	$\gamma^{c,ult}$	$G^{c}$
	(N)	(MPa)	(10^−3^)	(MPa)	(10^−3^)	(MPa)
Mean	2904 ± 513	5.16 ± 0.9	6.07 ± 1.1	813 ± 146	5.88 ± 1.2	816 ± 123
SD	673	1.18	1.43	192	1.54	162
COV	23.2	22.8	23.6	23.7	26.2	19.8

${}^{ult}$ corresponds to the maximum external force; ${}^{g}$ the values from the virtual gauges; ${}^{c}$ the values for central point of the cross-section.

**Table 3:** Material parameters for numerical simulations.

Criterion	Parameters		
Compression	$Y_{cL}$ (MPa)	$Y_{cR}$ (MPa)	$K_{c}$
	40.8	7.8	0.0
Tension	$Y_{tL}$ (MPa)	$Y_{tR}$ (MPa)	$K_{t}$
	80.5	3.8	0.0
Shear	$C_{LL}$ (MPa)	$C_{RR}$ (MPa)	μ
	6.0	4.5	0.1

## Data Availability

The data presented in this study are available on request from the corresponding author.
